# Cellular uptake and intracellular degradation of poly(alkyl cyanoacrylate) nanoparticles

**DOI:** 10.1186/s12951-015-0156-7

**Published:** 2016-01-08

**Authors:** Einar Sulheim, Habib Baghirov, Eva von Haartman, Andreas Bøe, Andreas K. O. Åslund, Yrr Mørch, Catharina de Lange Davies

**Affiliations:** Department of Physics, The Norwegian University of Science and Technology, NTNU, Høgskoleringen 5, 7491 Trondheim, Norway; SINTEF Materials and Chemistry, Trondheim, Norway; Pharmaceutical Sciences Laboratory, Faculty of Natural Sciences and Technology, Åbo Akademi University, Turku, Finland

**Keywords:** Poly(alkyl cyanoacrylate), Nanoparticles, Intracellular delivery, Degradation, Fluorescence lifetime

## Abstract

**Background:**

Poly(alkyl cyanoacrylate) (PACA) nanoparticles have shown promise as drug carriers both to solid tumors and across the blood–brain barrier. Efficient drug delivery requires both high cellular uptake of the nanoparticles and release of the drug from the nanoparticles. Release of hydrophobic drugs from PACA nanoparticles is primarily governed by nanoparticle degradation, and this process has been poorly studied at the cellular level. Here we use the hydrophobic model drug Nile Red 668 (NR668) to investigate intracellular degradation of PACA nanoparticles by measuring changes in NR668 fluorescence emission and lifetime, as the spectral properties of NR668 depend on the hydrophobicity of the dye environment. We also assess the potential of poly(butyl cyanoacrylate) (PBCA) and poly(octyl cyanoacrylate) (POCA) nanoparticles for intracellular drug delivery in the prostate cancer cell line PC3 and rat brain endothelial cell line RBE4 and the role of endocytosis pathways in PACA nanoparticle uptake in those cell lines.

**Results:**

Fluorescence lifetime imaging, emission spectra analysis and Förster resonance energy transfer indicated that the intracellular degradation was in line with the degradation found by direct methods such as gas chromatography and scanning electron microscopy, showing that PBCA has a faster degradation rate compared to POCA. The combined P(BCA/OCA) nanoparticles had an intermediate degradation rate. The uptake of POCA and PBCA nanoparticles was much higher in RBE4 than in PC3 cells. Endocytosis inhibition studies showed that both clathrin- and caveolin-mediated endocytosis were involved in PACA nanoparticle uptake, and that the former played a predominant role, particularly in PC3 cells.

**Conclusions:**

In the present study, we used three different optical techniques to show that within a 24-hour period PBCA nanoparticles degraded significantly inside cells, releasing their payload into the cytosol, while POCA nanoparticles remained intact. This indicates that it is possible to tune the intracellular drug release rate by choosing appropriate monomers from the PACA family or by using hybrid PACA nanoparticles containing different monomers. In addition, we showed that the uptake of PACA nanoparticles depends not only on the monomer material, but also on the cell type, and that different cell lines can use different internalization pathways.

**Electronic supplementary material:**

The online version of this article (doi:10.1186/s12951-015-0156-7) contains supplementary material, which is available to authorized users.

## Background

Achieving sufficient drug accumulation in the tumor and limiting toxicity towards healthy tissues are major challenges in cancer treatment [1]. One strategy is to encapsulate drugs into nanoparticles (NPs). Circulating NPs passively accumulate in some solid tumors due to fenestrations in tumor capillaries and lack of functional lymphatics, the so-called enhanced permeability and retention effect [2].

One of the advantages of NP-mediated drug delivery is the possibility of targeted, controlled and sustained release of drugs. In most target organs, cellular uptake of NPs occurs primarily through clathrin-mediated endocytosis (CME), although caveolin-mediated endocytosis (CavME) and other mechanisms can also play a role [3]. Intracellular drug release from the NPs occurs either by NP degradation or drug diffusion out of the NP, and can also be induced by external triggers such as hyperthermia [4], ultrasound [5] or changes in the local microenvironment, for instance pH [6].

Among various nanoparticles currently investigated for their potential in cancer treatment, polymeric NPs have emerged as promising drug carriers. Their advantages include easy fabrication and functionalization, biocompatibility, sustained drug release and controllable degradation rate [6]. Certain formulations based on PACA NPs have reached Phases II and III in clinical trials [7, 8]. Recently, we described the synthesis of poly(alkyl cyanoacrylate) (PACA) NPs using a one-step miniemulsion process [9]. These particles can be made from different alkyl cyanoacrylate monomers and their mixtures, leading to varying degradability.

Release of highly hydrophobic drugs from PACA NPs appears to be primarily governed by NP degradation. Therefore, uptake profile and intracellular degradation of PACA NPs are the two key factors affecting intracellular drug availability once the NPs have reached the tumor cells. The uptake of NPs in cells, including PACA NPs, demonstrates different uptake efficacy by different cell types and organs [10, 11]. Degradation of PACA NPs mainly occurs by surface erosion [12, 13] into water-soluble poly(cyanoacrylic acid) and a primary alcohol following hydrolysis of the ester [14]. This process can also be catalyzed by esterases [15]. Other proposed degradation mechanisms are probably less important at physiological conditions [16]. To the best of our knowledge, however, degradation and drug release for PACA NPs have been mainly studied in solution using physicochemical techniques [12, 17–19]. One recent study described the intracellular payload release from poly(butyl cyanoacrylate) NPs [11], but knowledge of the intracellular degradation of different PACA NPs remains scarce.

Thus, the aim of our work was to study the cellular uptake of PACA NPs and their intracellular degradation, leading to the release of a model drug. The cellular uptake and intracellular trafficking of PACA NPs were studied in two different cell lines using flow cytometry (FCM) and confocal laser scanning microscopy (CLSM). Rat brain endothelial cells (RBE4) were chosen because of the reported ability of PACA NPs to cross the blood brain barrier [20], and human prostate cancer cells (PC3) were chosen to assess NP uptake and degradation in a common human tumor cell line. Two different monomers, butyl cyanoacrylate (BCA) and octyl cyanoacrylate (OCA), were used to produce PBCA, POCA and P(BCA/OCA) NPs. PBCA NPs are reported to degrade faster than POCA, and their copolymer could potentially have a tunable, intermediate degradation rate that could be used to achieve required drug release kinetics [13].

Degradation rates under different extracellular conditions were compared to intracellular model drug release. Nile Red 668 (NR668), a novel hydrophobic dye with the emission spectrum depending on the hydrophobicity of the local environment, was chosen due to its high hydrophobicity and unique spectral properties [21]. In addition, NR668 shows no leakage out of our PACA NPs (Additional file 1: Figure S1), which is required to avoid false interpretation of results based on fluorescence signals. Both its emission spectrum and fluorescence lifetime depend on the local environment and can be used to locate the dye intracellularly. Emission spectrum analysis has previously been used by our group to locate Nile Red [22], while the use of fluorescence lifetime imaging (FLIM) to study intracellular degradation has, to our knowledge, only been reported for doxorubicin in poly(lactide-co-glycolide) NPs [23].

The three complementary optical techniques showed that the intracellular degradation rates of the PBCA and POCA NPs are in line with the rates measured in solution. In addition, we report that the uptake of PACA NPs depends both on the monomer material and the cell line used, and that different cells can use different internalization pathways for PACA NP endocytosis.

## Results

### Initial NP characterization

The diameters of the three NPs were in the range of 148–177 nm with a relatively narrow size distribution [polydispersity index (PDI) ≤ 0.12]. All three NPs were slightly negatively charged with a zeta-potential of approximately −10 mV. The molecular weight of the PBCA and POCA NPs was approximately the same 3500 and 3700 Da, respectively (Table 1).

**Table 1:** Physical parameters of the NPs

NP	Hydrodynamic diameter (nm)	PDI	ζ-potential (mV)	Polymer molecular weight (D)	Half-life at pH 7.4 ( h)
PBCA	177	0.12	−12	3500	25
POCA	151	0.10	−10	3700	48
P(BCA/OCA)	148	0.10	−9	–	∼500

### Cellular uptake of PACA nanoparticles

**Fig. 1:**
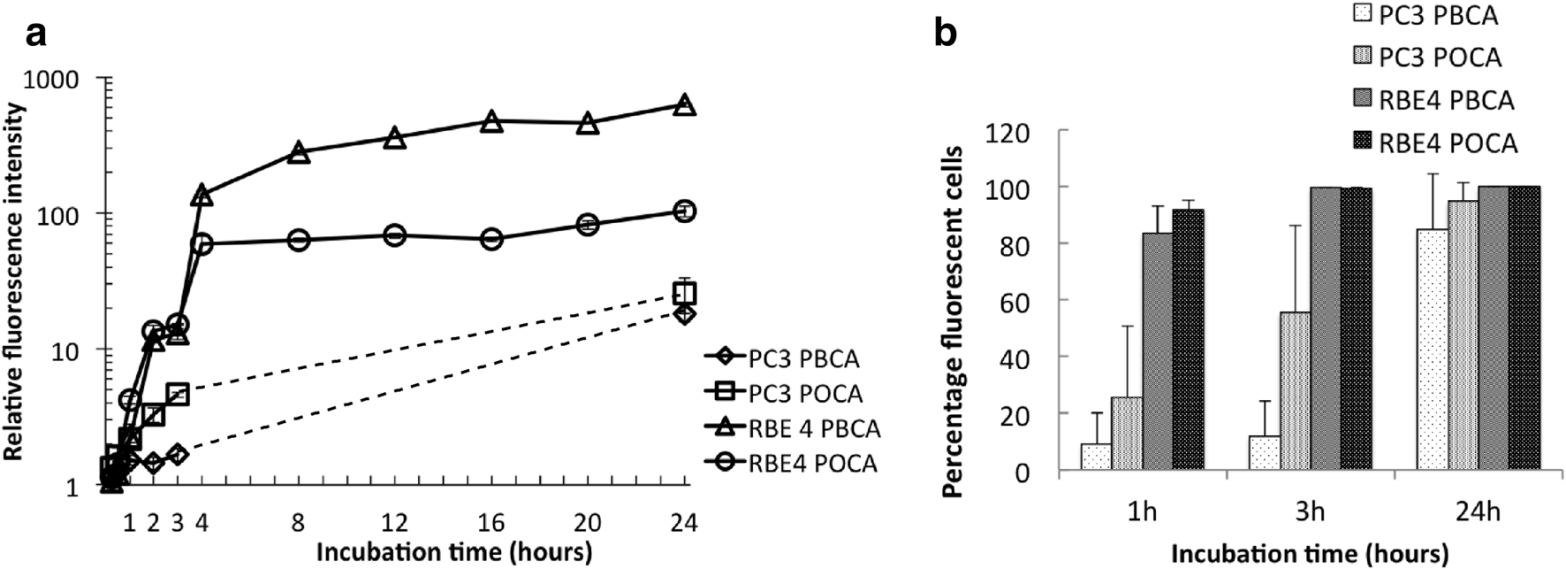
**a** PBCA and POCA NP uptake kinetics in PC3 and RBE4 cells expressed as fluorescence intensity relative to untreated cells on a logarithmic scale. **b** Percentage of NP-positive cells. n = 2, *error bars* are SD, partly within the *symbols*

**Fig. 2:**
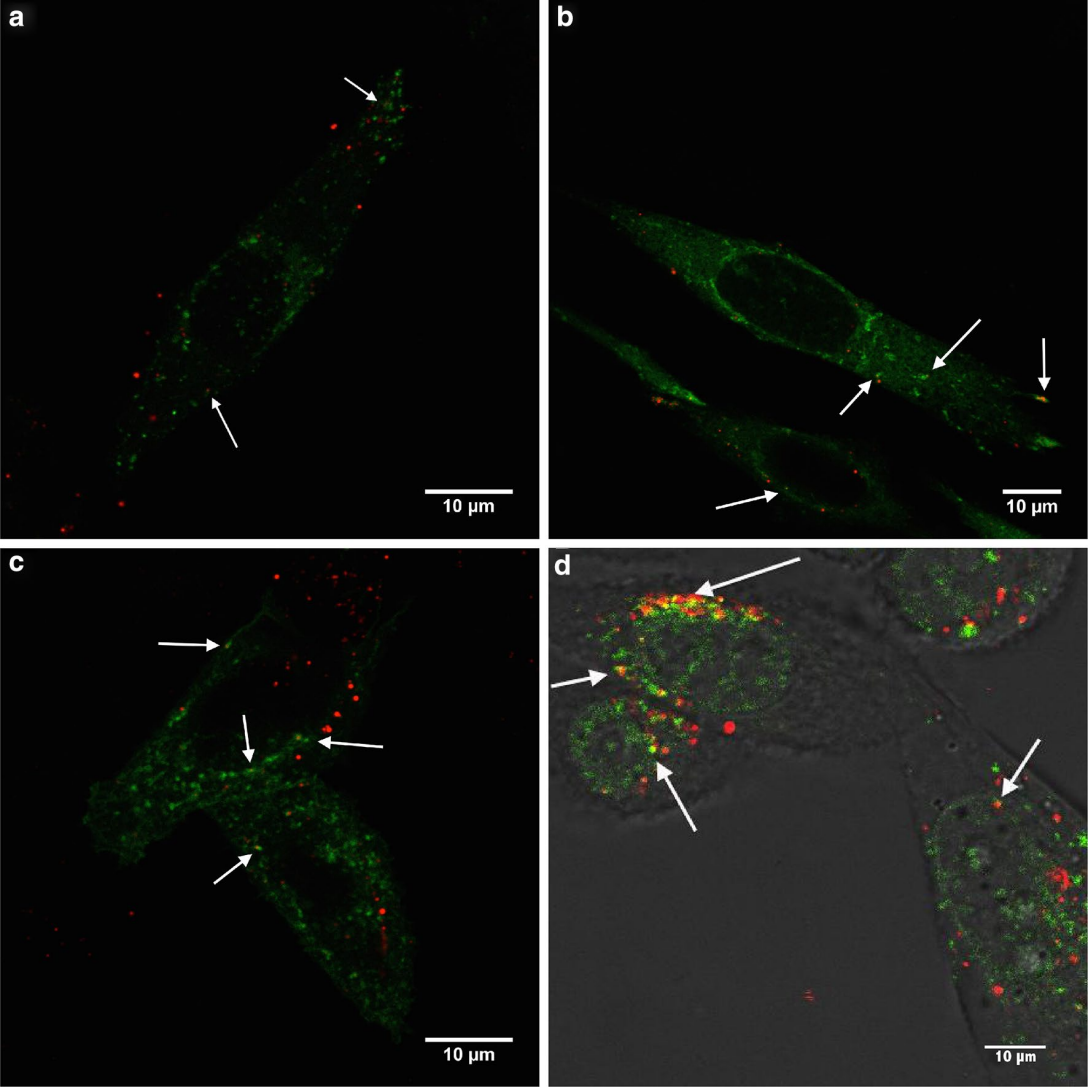
**a** Colocalization (*white arrows*) of PBCA NPs (*red*) with early endosomes), **b** late endosomes and **c** lysosomes (all in *green*) in RBE4 cells. **d** Colocalization of POCA NPs and lysosomes in PC3 cells. Both colors in the same pixel are seen as *yellow*. Cells were incubated with NPs for 3 h after labeling the endocytic compartments

PBCA and POCA NP uptake kinetics in PC3 and RBE4 cells were measured using FCM (Fig. 1) and internalization was confirmed by CLSM (Fig. 2). Cellular uptake is presented both as fluorescence intensity (Fig. 1a) and the percentage of fluorescent cells (Fig. 1b).

RBE4 cells had a significantly higher NP uptake than PC3 cells with more than fourfold difference after 3 h. After 24 h, PBCA and POCA NP uptake in RBE4 cells was 40 and 8 times higher, respectively, compared to PC3 cells. Approximately 90 % of RBE4 cells internalized NPs after 1 h, whereas PC3 cells reached this level after approximately 24 h (Fig. 1b). The cellular uptake depended on the monomer. In RBE4 cells, PBCA and POCA NPs were initially taken up with approximately equal efficiency, while sixfold higher uptake of PBCA NPs was observed after 24 h compared to POCA NPs. On the other hand, in PC3 cells the uptake of POCA NPs was eightfold higher than PBCA NPs already after 3 h (Fig. 1a).

PBCA NP colocalized with early endosomes, late endosomes and lysosomes in RBE4 cells (Fig. 2a, b, c) and POCA NP colocalized with lysosomes in PC3 cells (Fig. 2d). Both cell lines were incubated with NPs for 3 h and, assuming continuous internalization, some NPs were expected to be found in the various endocytic compartments. Figure 2 shows some colocalization (white arrows), but many endocytic compartments contained no NPs, and several NPs did not colocalize with any endosomes or lysosomes.

**Fig. 3:**
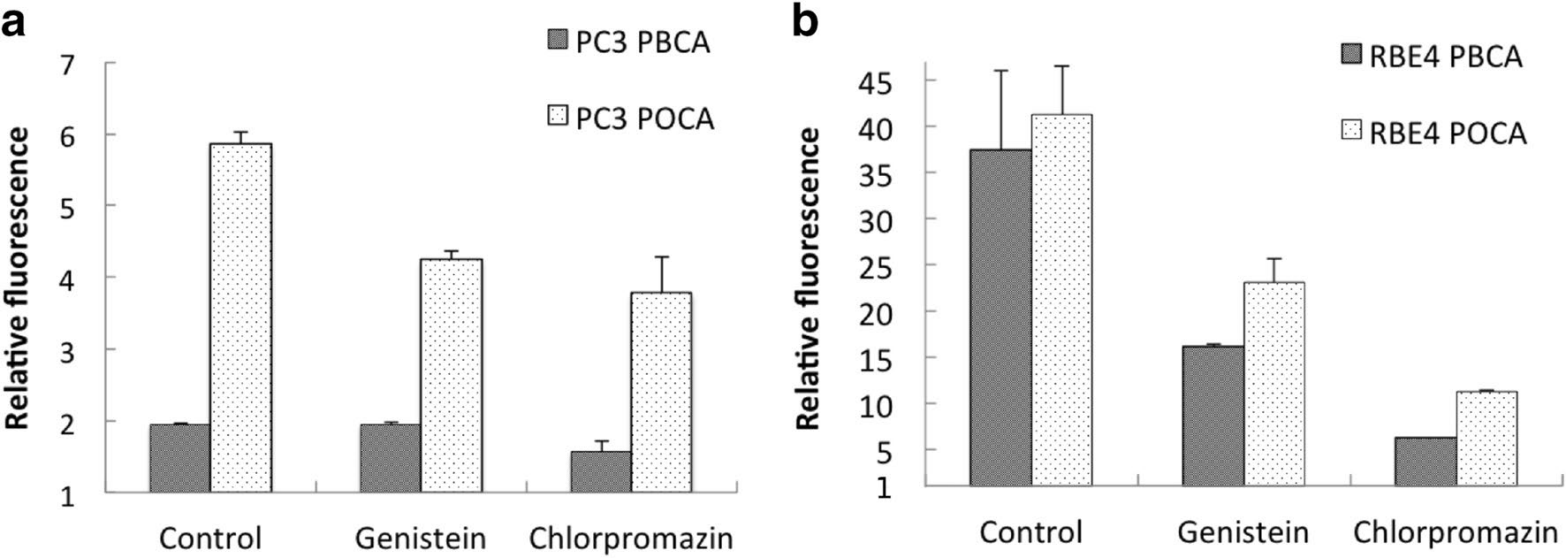
The effect of inhibition of endocytosis by genistein and chlorpromazine on the uptake of PBCA and POCA NPs in **a** PC3 cells and **b** in RBE4 cells. Control is untreated cells. The median fluorescence intensity is expressed relative to autofluorescence. n = 2, *error bars* give SD

Endocytosis inhibitors demonstrated that both CME and CavME were important uptake mechanisms (Fig. 3). In PC3 cells, both inhibitors reduced POCA NP uptake by approximately 40 %. Inhibiting CavME did not affect PBCA NP uptake in PC3 cells, whereas inhibiting CME reduced PBCA NP uptake by approximately 40 %. Endocytosis inhibition in RBE4 cells had a greater effect than in PC3 cells as inhibition of CME and CavME reduced POCA NP uptake by 73 and 43 %, respectively, and PBCA NP uptake by 83 and 56 %, respectively. Endocytosis is an energy-dependent process and is strongly inhibited at low temperatures [24]. Thus, the cells were incubated with NPs for minimum 2 h at 4 °C. Uptake was observed in neither PC3 nor RBE4 cells (Additional file 1: Figure S1).

### PACA NP degradation

**Fig. 4:**
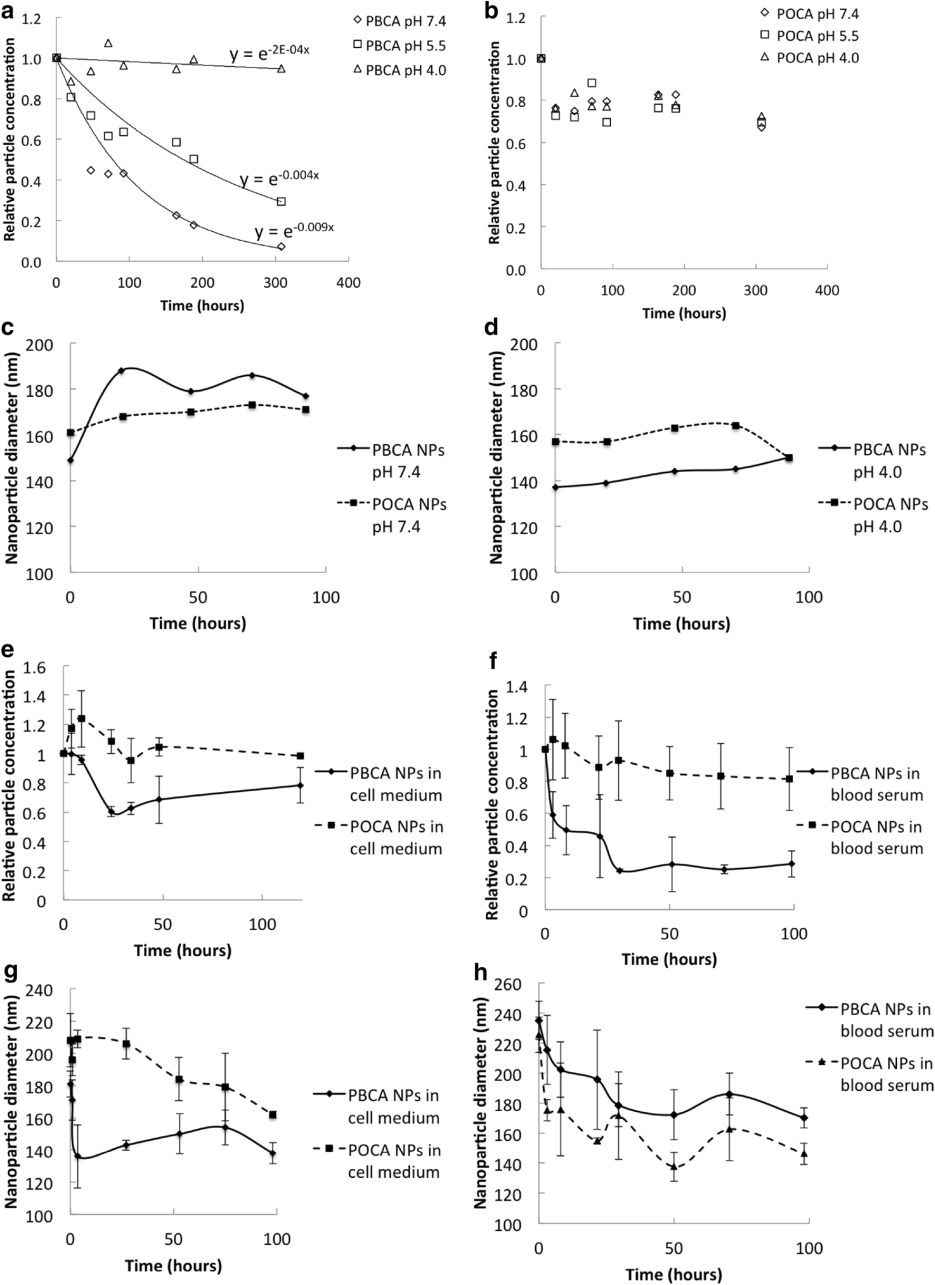
**a** Concentration of PBCA NPs and **b** POCA NPs at pH 7.4, 5.5 and 4.0. An exponential decay was fitted to the results in **a**. **c** Mean NP diameter at pH 7.4 and **d** pH4.0. Relative concentration (**e**, **f**) and mean diameter of NPs (**g**, **h**) in cell medium (**e**, **g**) and human blood serum (**f**, **h**)

To study degradation in physiological relevant solutions, buffers at different pH as well as cell medium and human blood serum were used, and NP size and concentration were measured using Nanoparticle Tracking Analysis (NTA) (Fig. 4). PBCA NPs were found to degrade both in buffers at neutral pH, in cell medium with serum and in human serum.

The relative concentration of PBCA NPs decreased exponentially with half-lives of 144 days at pH 4, 7 days at pH 5.5 and 3 days at pH 7.4 (Fig. 4a). POCA NPs showed little sensitivity to pH. At all pH tested, the concentration decreased by only 10–20 % within the first 20 h and no further decrease was observed (Fig. 4b). A significant increase in NP diameter was observed for PBCA NPs. At physiological pH, the particle size increased by 20–30 % after 20 h, but was almost unchanged at pH 4.0 (Fig. 4c, d). POCA NPs, however, only expanded by 4 % at pH 7.4, with no changes observed at pH 4.0 (Fig. 4c, d). Both in cell medium and human serum, PBCA NP concentration decreased significantly, while POCA NP concentration was barely affected (Fig. 4e, f). For both particles, a significant increase in the initial size was observed in both cell media and serum, probably due to swelling and the formation of a protein corona (Fig. 4f, g).

**Fig. 5:**
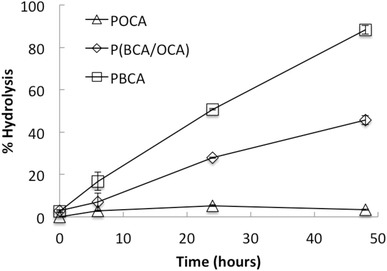
Degradation of PBCA, P(BCA/OCA) and POCA at pH 7.4 measured by gas chromatography. Degradation is indicated as  % hydrolysis of the NPs as a function of time. 100 % hydrolysis was obtained in glycine buffer at pH 9. n = 3, *error bars* give SD

More quantitative degradation experiments were performed in buffers at pH 7.4 using gas chromatography (GC) to measure the degradation products butanol and octanol. The degradation was measured as percentage of complete hydrolysis in glycine buffer at pH 9 (Fig. 5). After 48 h, 88 % degradation was found for PBCA NPs, as compared to only 3 % for POCA NPs, confirming much faster degradation of PBCA. Assuming a linear degradation rate, this gives a half-life of 25 h for PBCA NPs, which is 33 % of what was found using NTA (Fig. 4a; Table 1). However, while NTA measures degradation in terms of NP concentration, GC measures the amount of degradation products. For the copolymer P(BCA/OCA), an intermediate rate of degradation (45 %) was found after 48 h. For POCA, linear regression gave a poor fit, but indicated a half-life of approximately 500 h.

**Fig. 6:**
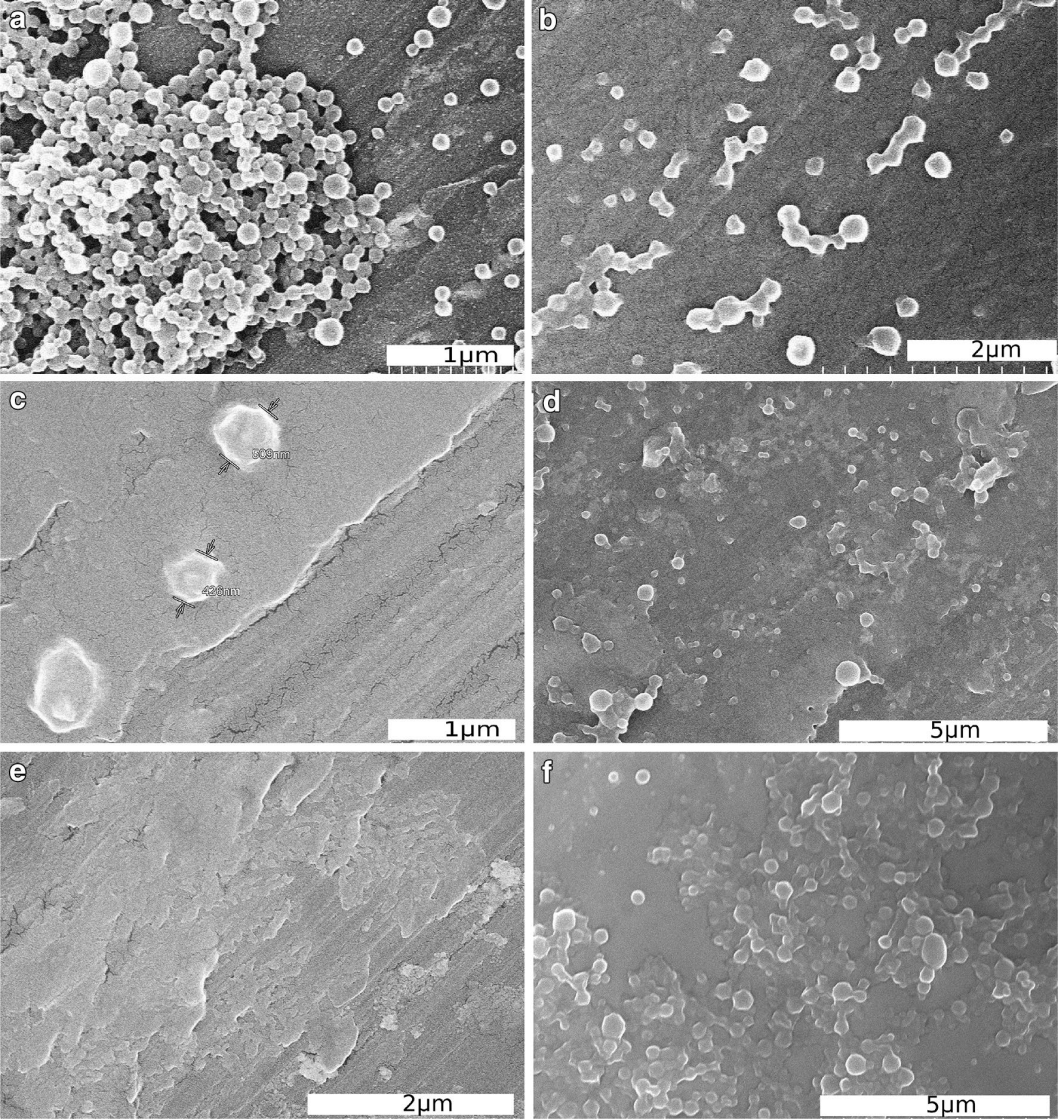
SEM images of PBCA (**a**, **c**, **e**) and POCA NPs (**b**, **d**, **f**) incubated at pH 7.4 for 0 days (**a**, **b**), 8 days (**c**, **d**) and 11 days (**e**, **f**)

To visualize the degradation, NPs were imaged by scanning electron microscopy (SEM) after 0, 8 and 11 days of incubation at pH 7.4 (Fig. 6). On day 0 intact, spherical NPs were observed. After 8 and 11 days, the POCA NPs were still intact (Fig. 6d, f) whereas no PBCA NPs were seen, probably due to degradation of the NPs (Fig. 6c, e).

**Fig. 7:**
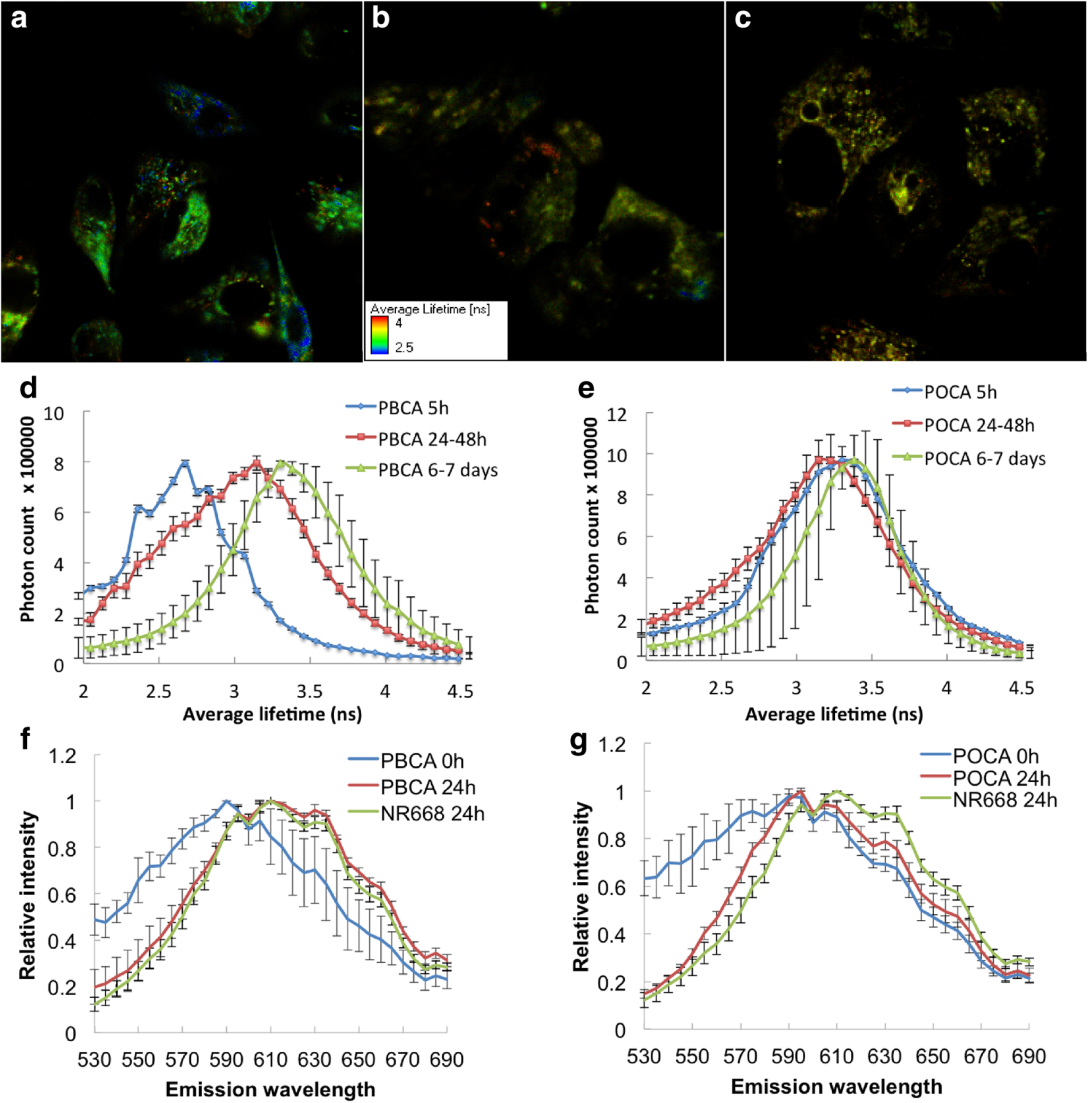
**a**–**c** FLIM images of PC3 cells incubated for 24 h with free NR668 (**a**), PBCA (**b**) and POCA NPs (**c**) before growing for additional 5 days. Each pixel is colored based on the average lifetime. Fluorescence lifetime distribution for PBCA (D) and POCA (E) NPs inside cells at 5, 24–48 and 144–168 h (6–7 days). Emission spectra from PC3 cells incubated with free NR688 (*green*) or PBCA (**f**) or POCA NPs (**g**) after 24 h (*red*), or the NPs prior to incubation (*blue*). n = 5, *error bars* give SD

Intracellular degradation of the NPs was studied by measuring the release of NR688 using the spectral properties of the dye. NR668 appeared to be strongly associated with NPs as no cellular fluorescence was seen at 4 °C, and no diffuse cytoplasmic staining was observed in CLSM in agreement with [21]. Thus, any labeling of cytoplasmic lipid droplets or hydrophobic molecules should be caused by NR668 being released from degraded NPs. The size and distribution of lipid droplets are often visually similar to those of NPs in endosomes or lysosomes, and the dye location is therefore difficult to evaluate using CLSM [25]. FLIM and spectral analysis were used to study whether the dye was inside intact NPs or associated with other hydrophobic cellular molecules. FLIM images of PC3 cells incubated with free NR668, PBCA NPs and POCA NPs are shown in Fig. 7a, b, c. The cells were incubated with NR668-loaded NPs or the free dye for 24 h before removing the dispersion and growing the cells for additional 5 days. The images show multiple lifetimes, from approximately 2.5 ns (blue) to 4 ns (red) in cells incubated with the free dye (Fig. 7a) and PBCA NPs (Fig. 7b), whereas a narrow lifetime distribution around 3 ns (yellow–green) was seen in cells incubated with POCA NPs (Fig. 7c).

To further quantify the variations in fluorescence lifetime, its distribution was measured in cells incubated with PBCA (Fig. 7d) or POCA NPs (Fig. 7e). In cells incubated with PBCA NPs, the fluorescence lifetimes increased significantly with time while the POCA NP lifetimes remained almost unchanged, indicating that even after 6–7 days inside the cells, POCA NPs were still intact while PBCA NPs were continuously degrading. However, 77 % of the change in lifetime (0.7 nsec) occurred already after 24–48 h. The large standard deviations (SDs) for POCA NPs after 6–7 days (Fig. 7e) indicate that those particles also underwent some changes. Figure 7d, e show the average of the long and short lifetimes, and the correlation between the two lifetimes is found in Additional file 1: Figure S2.

The fluorescence emission spectra were recorded for cells incubated with both PBCA and POCA NPs for 24 h, and compared with those of free NR668 incubated with cells for 24 h as well as NPs prior to incubation (Fig. 7f, g). After 24 h, the emission spectrum of PBCA shifted to higher wavelengths and became similar to that of free NR668 inside cells. However, the emission spectrum of POCA NPs only shifted slightly towards the spectrum of free NR668, and was similar to the spectrum of POCA NPs not incubated with cells.

**Fig. 8:**
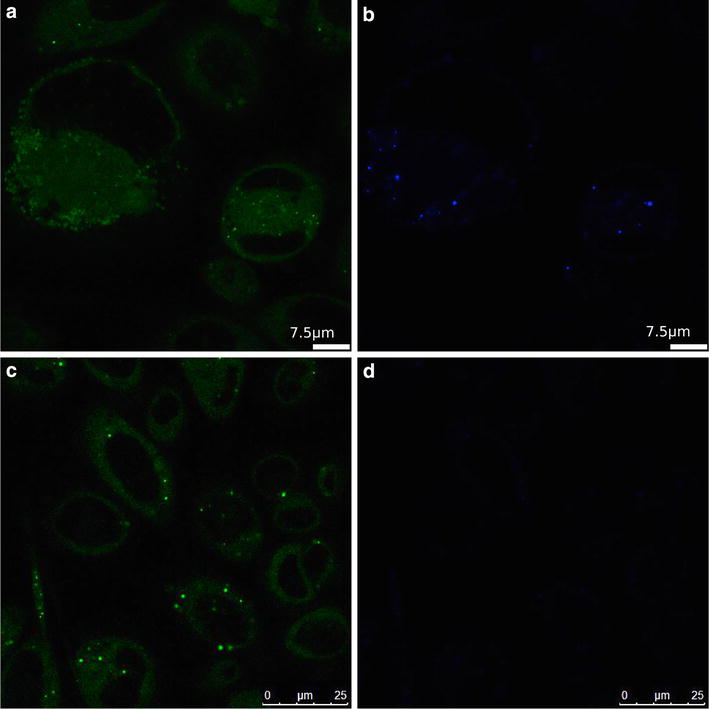
PC3 cells incubated with PBCA (**a**, **b**) or POCA NPs (**c**, **d**) for 24 h and grown for additional 72 h before p-HTAM staining. Excited at 405 nm and p-HTAM fluorescence detected at 500–540 (**a**, **c**) and FRET signal at 650–710 nm (**b**, **d**)

To confirm that the changes in fluorescence lifetime and emission spectra were due to dye release from the NPs and not changes in the environment inside the NPs, we used pentamer hydrogen thiophene acetic acid methyl ester (p-HTAM), a hydrophobic dye that forms a Förster resonance energy transfer (FRET) pair with NR668. PC3 cells were incubated with POCA and PBCA NPs for 24 h and then grown for 3 days in medium without NPs. One hour prior to CLSM-imaging, the cells were stained with p-HTAM. p-HTAM was excited at 405 nm and detected in the interval of 500–540 nm (Fig. 8a, c). A FRET signal from NR668, detected at 650–710 nm was observed in cells incubated with PBCA NPs (Fig. 8b), but not with POCA NPs (Fig. 8d), demonstrating that NR668 from PBCA was located in close proximity to the p-HTAM molecules.

## Discussion

### Cellular uptake

PACA NPs are promising carriers for drugs [26] and oligonucleotides [27] both across the blood–brain barrier [28] and to cells in solid tumors [22]. Endocytosis is the predominant cellular uptake mechanism, although contradictory results have been reported. Earlier studies showed no endocytosis [29] whereas others [30, 31] found that PACA NP uptake was an energy-dependent process as no uptake occurred at 4 °C, similarly to the results presented here. The discrepancy could be explained by the production methods that have been shown to drastically change the interaction between cells and NPs [32]. The lack of cellular fluorescence at 4 °C also demonstrated that NR668 was not leaking out of PACA NPs or taken up by the cells through NP-cell contact-mediated transfer, as reported earlier for Nile Red [25].

Endocytosis efficiency was cell type-dependent, and RBE4 cells originating from rat brain endothelium demonstrated efficient uptake. The prostate tumor cell line PC3 showed a slow uptake and the amount of NPs per cell was 10–40 times lower than in RBE4 cells. A cell type-dependent uptake of NPs has previously been reported for gold NPs [33], chitosan NPs [34], and polystyrene NPs of various sizes [35]. In the latter work, approximately tenfold higher uptake of NPs was observed in human brain endothelial cells (HCMEC D3), as compared to cervical cancer cells (HeLa) and human lung epithelium cells (A549). Thus, the high uptake in RBE4 cells might be a property of brain endothelial cells, and in accordance with this, PACA NPs are reported to cross the blood–brain barrier when coated with certain polyethylene glycol (PEG) surfactants [20, 36].

CME and CavME inhibition showed that both endocytic pathways were active. CME seemed to be the dominant mechanism, as its inhibition more strongly affected cellular uptake in both cell lines, especially in RBE4 cells, but also PBCA uptake in PC3 cells. For RBE4 cells, the combined inhibition effect of CME and CavME inhibitors exceeded 100 %, indicating poor inhibition selectivity. For PC3 cells, the inhibition effect was lower, with possible contribution of other pathways. CavME inhibition did not affect PBCA NP uptake in PC3 cells, showing that CME represents the predominant mechanism of PBCA NP uptake. To study intracellular trafficking further, early endosomes, late endosomes and lysosomes were visualized, but only limited colocalization with those organelles was observed for both POCA and PBCA NPs. This indicates that NPs may escape the endocytic pathway or that endocytosis was not a continuous process in the studied timeframe. The mechanism of possible escape from the endocytic pathway is unclear, but other groups have also observed limited colocalization with lysosomes for polymeric NPs [27, 37].

POCA NPs were internalized more efficiently than PBCA NPs in PC3 cells, whereas the opposite was true for RBE4 cells. Two known NP properties that determine endocytosis are size and charge [34]. Lower uptake with increasing NP size has been reported for CME and CavME [38]. The size and charge of POCA and PBCA NPs were not very different when measured by dynamic light scattering (DLS) in phosphate buffer. However, incubation in cell medium for several hours could change the NPs, especially the PBCA NPs that started degrading immediately upon incubation. We have seen that PBCA degradation starts with significant enlargement, probably due to swelling [39]. Both NPs expanded instantly in serum-supplemented cell medium and human serum, likely due to both swelling and protein corona formation [40]. However, the increase in size was rather small for both NPs and does probably not fully explain the differences in internalization.

Cationic NPs are internalized more efficiently than anionic NPs [34]. Surface degradation of PACA NPs leads to the formation of carboxylic acid, making the surface more hydrophilic [41, 42]. We have found that the NP zeta-potential decreases with time in medium, where PBCA NPs became more negatively charged than POCA NPs (unpublished results). This observation might explain the higher uptake of POCA NPs in PC3 cells. However, in RBE4 cells the PBCA NPs were internalized much more efficiently. Several other studies also demonstrate a high uptake of PBCA NPs in brain endothelial cells [36] whereas, to our knowledge, POCA NP uptake by brain endothelium has not been reported. It is also noteworthy that RBE4 cells use both CME and CavME, whereas PC3 cells mainly use CME, especially for PBCA NPs, which may partly explain the observed differences between RBE4 and PC3 cells.

### Degradation

NP degradation rate is crucial in terms of circulation time, stability, toxicity and drug release rate. The various degradation rates of the PACA family polymers have been shown to affect drug release rates [43]. Drug delivery through biodegradation enables continuous delivery, as opposed to burst delivery in conventional chemotherapy. Thus, depending on the alkyl monomer chain length [12] and polymerization mechanism [44], PACA NPs may be made to continuously release the drug intracellularly for weeks, potentially affecting slowly proliferating or quiescent cancer cells. Degradation in biological media is influenced by pH, but also by enzymatic degradation from esterases [16, 17]. In order to investigate the contribution of esterase-mediated degradation, esterase activity was monitored in serum-supplemented growth medium and in human serum over 96 h. No changes in esterase activity were observed that could explain the differences in PBCA and POCA NP degradation rates (Additional file 1: Figure S3). Esterase activity was reduced instantly and recovered within 24 h. Thus low degradation of NPs after approximately 48 h could not be attributed to loss of esterase activity.

In the present work, NP degradation is apparently required for NR668 release, as the dye is closely associated with the polymeric network of the NP through hydrophobic interactions. This was demonstrated by the correlation between the measured degradation rate of the NPs in physiological solutions and the release of NR688 from the NPs measured intracellularly. After 48 h, 88 % of the PBCA NPs had degraded as measured with GC. Similarly, 77 % of the change in fluorescence lifetime measured intracellularly had occurred at this time point. NPs were stable at low pH, but degraded rapidly at pH 7.4. The concentration of POCA NPs was still close to 80 % after over 300 h at pH values of 4.0–7.4. The degradation observed with NTA was confirmed by GC and SEM. Furthermore, GC showed that the degradation rate could be manipulated by mixing the two monomers.

Intracellular degradation of PBCA NPs was also more efficient than that of POCA NPs. Release of NR668 occurred from PBCA, but hardly from POCA NPs. This was demonstrated intracellularly by FLIM and by the spectral changes of the dye. Upon degradation, the fluorescence lifetime for PBCA NPs increased towards the lifetime for POCA NPs. This might be because released NR668 diffuses to areas of higher hydrophobicity such as lipid droplets, thereby reaching lifetimes similar to intact POCA NPs. The emission spectra of NR668 in cells incubated with PBCA NPs showed that after 24 h the dye environment resembled that of free NR668. Furthermore, colocalization with p-HTAM also indicated a location different from endosomes/lysosomes or inside NPs, because p-HTAM enters the cell passively rather than by endocytosis. FRET requires that the fluorescent molecules be in close proximity (approximately 10 nm), making FRET impossible with the dye still encapsulated in the NPs [45]. Thus, the FRET signal observed in cells incubated with PBCA NPs, but not in cells with POCA NPs demonstrated that PBCA, but not POCA NPs were degraded. It has also been reported by others that PACA NPs with longer alkyl chains degrade more slowly [46, 47].

The bulk of our knowledge on PACA degradation originates from earlier physicochemical characterization studies [12, 17]. PACA NP-assisted drug delivery in vivo can also be indirectly linked to degradation rates. To the best of our knowledge, however, [11] remains the only work where the fate of PBCA nanocapsules was observed intracellularly using dual labeling of the nanocapsule shell and the cargo. That study, however, was performed with only one type of PACA NPs and mostly concerned the release and intracellular fate of the payload, while in the present work we studied the intracellular fate of different PACA nanocarriers.

Several groups have suggested pH-sensitive nanocarriers for drug delivery [48], increasing the degradation rate at lower pH. Our results suggest an opposite mechanism which is consistent with other results for PBCA NPs [15]. The NPs remain intact at acidic pH and the drug is released into the cytosol after NP escape from the lysosomal pathway, limiting lysosomal inactivation and exocytosis of the drug or NPs. For therapeutic effect, cytosolic release of drugs and therapeutics is reported to be far superior to lysosomal release [37].

NR668 release from PACA NPs was studied using three different optical methods. Emission spectrum analysis and FRET are well-established methods used to characterize free dye vs. intact NPs in tumor cells and tissue [22, 49]. FLIM is a novel method for studying intracellular degradation and, albeit relatively time-consuming, shows promise for probing the microenvironment of the delivered dye as it is very sensitive and less dependent on fluorophore concentration than emission spectrum analysis [50]. Since fluorescence lifetime is sensitive to hydrophobicity and pH [51], it was found to be a well-suited method for studying the release of the hydrophobic NR668 dye. Therefore, the combination of those three methods—emission spectrum analysis, FRET and FLIM, each providing complementary results,—allows reconstructing a comprehensive picture of intracellular degradation of PACA NPs and the release of a hydrophobic drug.

## Conclusions

In the present study, we showed that the uptake of PACA NPs depends not only on the monomer material, but also on the cell type, and that different cell lines can use different internalization pathways. Within a 24-hour period, PBCA NPs degraded significantly inside cells, releasing their payload into the cytosol, while POCA NPs remained intact. This shows that it is possible to tune the drug release rate by choosing appropriate monomers from the PACA family.

## Methods

### Synthesis and characterization of nanoparticles

The NPs used were either PBCA, POCA or P(BCA/OCA). All NPs were prepared using the miniemulsion polymerization method as described previously [9]. An oil-in-water emulsion was prepared by mixing a monomer oil phase with a water phase containing the non-ionic PEG stabilizer (Brij^®^L23, Sigma-Aldrich, 20 mM) in 0.1 M HCl. The monomer phase contained either butyl-2-cyanoacrylate (BCA, Henkel Loctite) or octyl cyanoacrylate (OCA, Henkel Loctite) or a mixture of the two, a neutral oil as co-stabilizer (Miglyol 810N, 2 wt %, Cremer), a radical initiator (V65, Azobisdimetyl valeronitril, Wako, 0.9 wt %) and 0.2 wt % of the fluorescent dye NR668 (modified Nile Red, a kind gift from Dr. Klymchenko, University of Strasbourg). After emulsifying (Branson Digital Sonifier, 60 % amplitude, 3 min), Jeffamin^®^M-2070 (a polyetheramine with a 19-unit PEG chain, Huntsman Corporation, 68 mM), was added to initiate the polymerization. The polymerization was carried out at room temperature overnight and at 50 °C for 8 h to activate the radical initiator for polymerization of remaining internal monomer. Spontaneous polymerization was controlled by performing emulsification at acidic conditions (0.1 M HCl). The particles were rinsed by extensive dialysis against 0.001 M HCl using dialysis membranes with MWCO 12–14,000. The synthesized NPs were characterized for size, PDI and surface charge (zeta-potential) using electrophoretic and dynamic light scattering (DLS, Zetasizer Nano ZS, Malvern Instruments) in 0.01 M phosphate buffer, pH 7. Molecular weight (MW) analysis by size exclusion chromatography was provided by Innventia AB, Sweden. Briefly, the NPs were dissolved in tetrahydrofuran and run through the three columns containing Styragel HR4, Styragel HR2 and Styragel HR1 at 0.8 ml/min. Polystyrene standards with MW from 1000 to 350,000 was used for calibration.

### Cell culture

Human PC3 prostate adenocarcinoma cells (CRL-1435, American type culture collection) were cultured at 37 °C and 5 % CO_2_ in Dulbecco’s Modified Eagle’s Medium (DMEM, Life Technologies Corporation, USA) supplemented with 10 % fetal bovine serum (FBS).

RBE4 cells (a generous gift from Dr. Aschner, Vanderbilt University) were cultured on collagen at 37 °C and 5 % CO_2_ in Ham’s F-10 medium supplemented with 10 % FBS, 300 µg/ml G418 and 1 ng/ml basic fibroblast growth factor (all from Life Technologies Corporation).

### Incubation with NP and inhibition of endocytosis

Cells (PC3 or RBE4) were seeded in 8-well plates (Ibidi) at 18,500 cells/well and cultured for 3 days when studying cellular uptake. NPs were added at a concentration of 20 µg/mL in growth medium and incubated for various times up to 24 h before changing the growth medium prior to imaging. For the degradation studies, 10,000 cells were seeded per well, the NPs were removed after 24 h and the cells were grown for additional 6 days changing medium every other day.

For endocytosis inhibition studies, PC3 or RBE4 cells were seeded in 12-well plates (Costar) at a density of 125,000 cells/well and grown to reach the log phase. Chlorpromazine was used as a specific inhibitor of CME [3]. Genistein is traditionally used to inhibit CavME, although some inhibition of CME subtypes has also been reported [52]. Chlorpromazine and genistein were added at 10 µg/mL and 13.5 µg/mL, respectively, as used by others [53, 54]. The inhibitors were pre-incubated with cells for 1 h before adding a dispersion containing NPs at 20 μg/ml together with the inhibitor, and incubating at 37 °C for 3 h. To achieve complete inhibition of endocytosis, the cells were pre-incubated for 5 min at 4 °C before adding NPs and incubating the cells at 4 °C for 2 or 3 h, in the case of RBE4 and PC3 cells, respectively.

### CLSM and cell labeling

Confocal images where obtained using a Leica SP8 CLSM with a 63 × 1.2 water objective. For NR668 excitation, a white light laser at 514 nm was used, and emission was detected at 580–660 nm using a photon counting hybrid detection system. Z-stacks of cells were obtained to verify that the NPs were intracellular.

In PC3 cells, lysosomes were stained with the pH sensitive dye LysoTracker Blue (DND-22, Life Technologies) at 2.5 μM for 1 h and imaged using a pulsed multiphoton laser at 780 nm to excite the dye. In RBE4 cells early endosomes, late endosomes and lysosomes were labeled by cellular transduction using CellLight Early Endosomes-GFP, CellLight Late Endosomes-GFP and CellLight Lysosomes-GFP (Life Technologies), respectively, at a concentration of 40 particles per cell 24 h prior to imaging. After labeling the organelles, cells were incubated with NPs for 3 h before imaging.

### Flow cytometry

The cells were analyzed using a flow cytometer (Gallios, Beckman Coulter) exciting at 561 nm and detecting at 630 nm with a 30 nm bandpass filter. Prior to FCM, the cells were washed three times in PBS. 10,000 cells were included in each sample, and a dot plot of forward light scatter signal versus side scatter signal was used to establish a collection gate that excluded cell debris, dead cells and aggregates. The cellular uptake of NPs was estimated both as the percentage of cells with NPs and as the amount of NPs per cell which was estimated from the median fluorescence intensity. To compare the uptake of PBCA and POCA NPs, which had different fluorescence intensity, a normalization factor of 1,9 (PBCA vs POCA) was used. This factor was found by measuring the fluorescence intensity using a spectrophotometer (Infinite 200Pro, Tecan). Thus the increase in median fluorescence between cells incubated with POCA NPs and the control cells was multiplied by this normalization factor and added to the autofluorescence.

### Emission spectra analysis

The emission spectra were obtained using the Leica SP8 CLSM. Intracellular NR688 was excited using a white light laser at 514 nm and detected in intervals of 10 nm from 550 to 700 nm. The intervals had an overlap of 5 nm resulting in 30 intervals. The spectra were normalized to the same maximum intensity for analysis.

### Flim

FLIM was recorded using a Leica SP8 microscope equipped with the PicoQuant system. NR668 was excited by a white light laser at 514 nm, with detection at 565–615 nm using a Single Photon Avalanche Detector. SymPhoTime PicoQuant was used to record lifetimes in time domain. The lifetimes τ_i_ were calculated using a two-exponential decay with the instrument response function E(t) that was fitted to the fluorescence decay curve:

$$F\left( {r,t} \right)\; = \;E\left( t \right)\; \times \;\mathop \sum \limits_{i = 1}^{2} A_{i} (r)e^{{ - \frac{t}{{\tau_{i} (r)}}}} .$$

The lifetimes were recorded until reaching a maximum intensity of 1000 photons/pixel. NR668 shows a two-exponential decay, resulting in two different lifetimes which were averaged using the equation:

$$\tau_{ave} = \frac{{\alpha_{1} \tau_{1}^{2} + \alpha_{2} \tau_{2}^{2} }}{{\alpha_{1} \tau_{1} + \alpha_{1} \tau_{1} }}$$

The software SymPhoTime provided by PicoQuant was used.

### Förster resonance energy transfer (FRET)

FRET was performed to verify that NR668 was physically removed from the NPs upon degradation. The cells were incubated with 20 µg/mL of PBCA or POCA NPs for 24 h and grown for 3 days before staining for 1 h with 1 µg/mL p-HTAM, a hydrophobic stain labeling, among others, lipid droplets [55]. NR668 released from the NPs might colocalize with p-HTAM in lipid droplets or other hydrophobic domains, enabling FRET. Images were obtained by exciting p-HTAM at 405 nm using a pulsed Hg-laser and detecting the FRET signal at 650–710 nm.

### NP degradation in solutions

Nanoparticle tracking analysis (NTA, Nanosight LM10-HS, Malvern Instruments) was used to determine NP concentration and diameter in various solutions. pH dependent NP degradation was studied in buffers with pH 7.4 (0.01 M phosphate buffer), pH 5.5 and 4.0 (0.01 M acetic acid). Degradation was also measured in cell medium (DMEM, Life Technologies) with FBS, pH 7.5 and human blood serum (a kind gift from professor Asbjørn Nilsen, Medical Faculty, NTNU). The NPs were added to the buffer at 20 µg NPs/ml and incubated for up to 336 h (14 days) at 37 °C. For cell medium and human serum, NPs were added at 5 mg/ml, and further diluted to 20 µg/ml in deionized water before NTA analysis. The buffer or medium were not changed during the incubation.

### Gas chromatography

Particles were diluted to 0.1 mg NP/ml in 0.01 M phosphate buffer pH 7.4 and kept at 37 °C with slow shaking. At various time points, the particles were centrifuged using an ultracentrifuge (WX Ultra 80, Thermo Electron Corporation) at 30,000 rpm for 2 h. For complete degradation, NPs were diluted to 0.1 mg NPs/ml in glycine buffer at pH 9 (0.2 M) and kept at 37 °C while shaking for 72 h before separation by ultracentrifugation. 3 µl of the internal standard *n*-pentane and 5 ml of diethyl ether were added to 5 ml of the supernatant and vortexed. 1 ml of the organic phase was analyzed with a GC Agilent 7890A equipped with a RTX-1 capillary column and helium as carrier gas. 1 µl of the sample was injected in split mode (1:5) and run isothermally at 45 °C for 3 min before initiating a temperature gradient of 10 °C/min until 200 °C. Pure butanol, pentanol and octanol samples were used as references.

### Scanning electron microscopy

For SEM, the NP-dispersion was diluted to 50 µg/ml in water and one droplet was placed on a SEM sample holder. After water evaporation, the samples were sputter-coated with a 10 nm gold layer and transferred to a Hitachi S-5500 at 15 kV acceleration voltage detecting secondary electrons.

## Additional file

10.1186/s12951-015-0156-7 Additional material.
