# Supplementary material for: Cellular uptake and intracellular degradation of poly(alkyl cyanoacrylate) nanoparticles
Source: J Nanobiotechnology. 2016 Jan 8;14:1. doi: 10.1186/s12951-015-0156-7 (PMC4705582; doi:10.1186/s12951-015-0156-7)
Supplement: Supplementary file 1 — 10.1186/s12951-015-0156-7 Additional material. [file 12951_2015_156_MOESM1_ESM.docx]

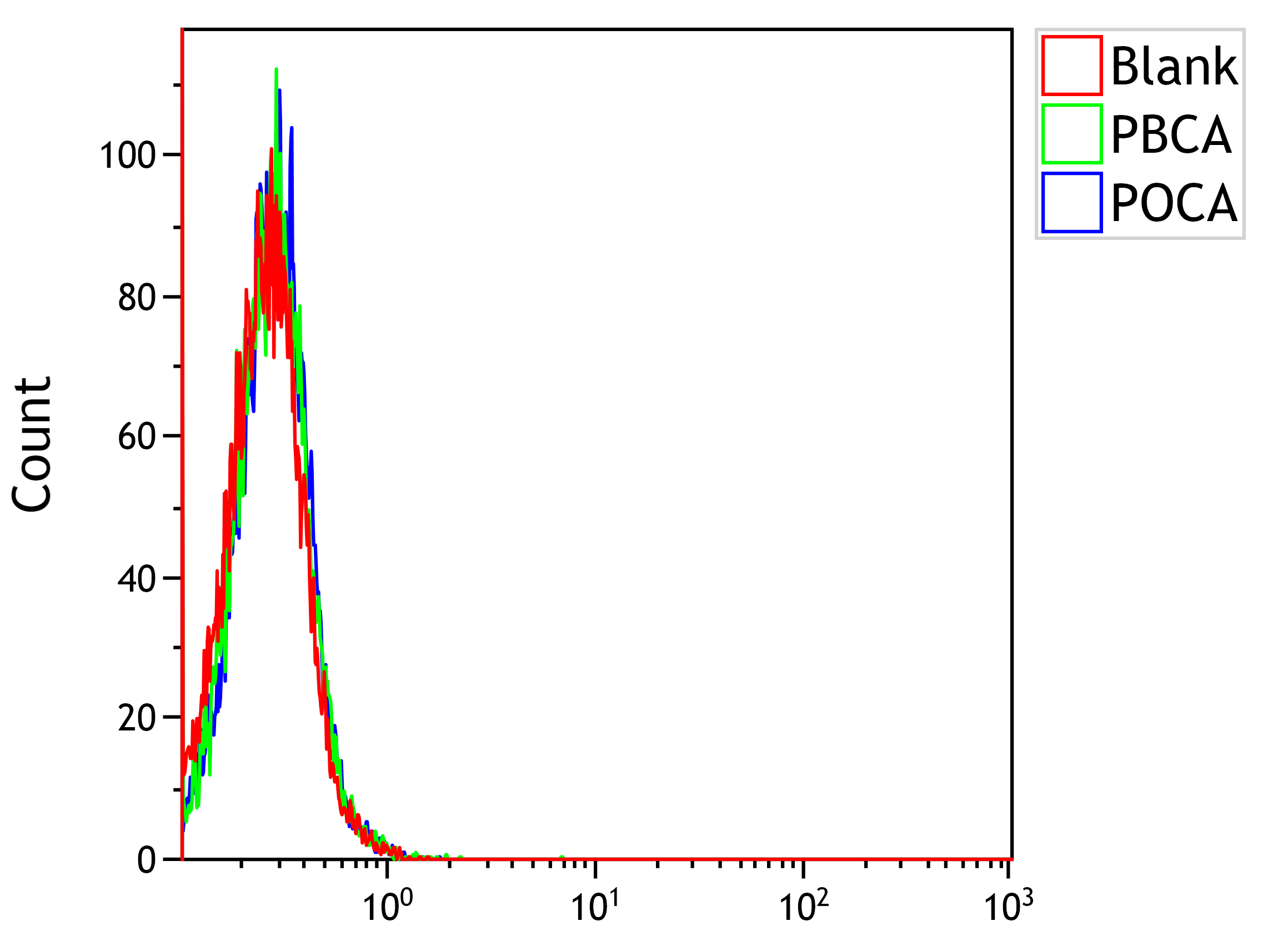


S1: RBE cells incubated for 2 hours with PBCA (green) and POCA(blue) at 4°C. Control population in red.


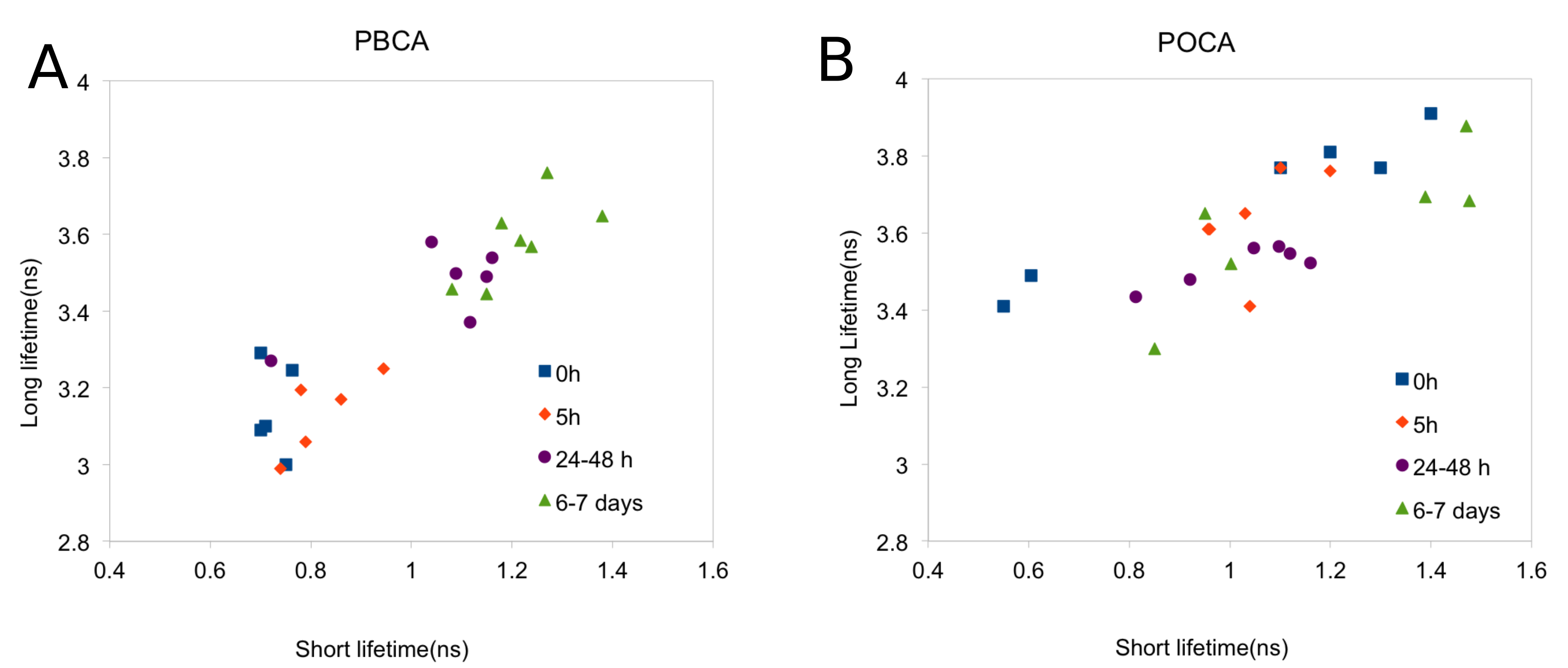


S2: The short (x-axis) and long (y-axis) lifetime found in FLIM images of PBCA (A) and POCA (B) NPs incubated with PC3 cells for various times.

S3: Esterase activity

*Materials and method:* NPs were dispersed in Dulbecco's Modified Eagle's Medium (DMEM, Life Technologies Corporation, USA) supplemented with 10% fetal bovine serum or in human serum. At t=0, 30 min, 24 hours, 48 hours 72 hours and 96 hours a sample was removed and the fluorescent substrate for esterase, 4-Methylumbelliferyl butyrate (Sigma Aldrich) was added to a final concentration of 2 μM. The fluorescence was recorded using a spectrophotometer (Tecan) exciting at 365 nm and detecting at 450 nm. Maximum fluorescence was measured by hydrolyzing the ester in glycine buffer at pH 9.

*Results:* A modest decrease in esterase activity was seen after 30 min in all samples as seen in figure S3. After 24 hours the esterase activity had returned to the initial values and the fluorescence was approximately 50 and 60 percent of complete hydrolysis for human serum and cell medium respectively. The esterase activity was the same in medium with 10% serum containing PBCA or POCA NPs and in human serum with PBCA or POCA NPs. The esterase activity did not decrease with time up to 96 hours.


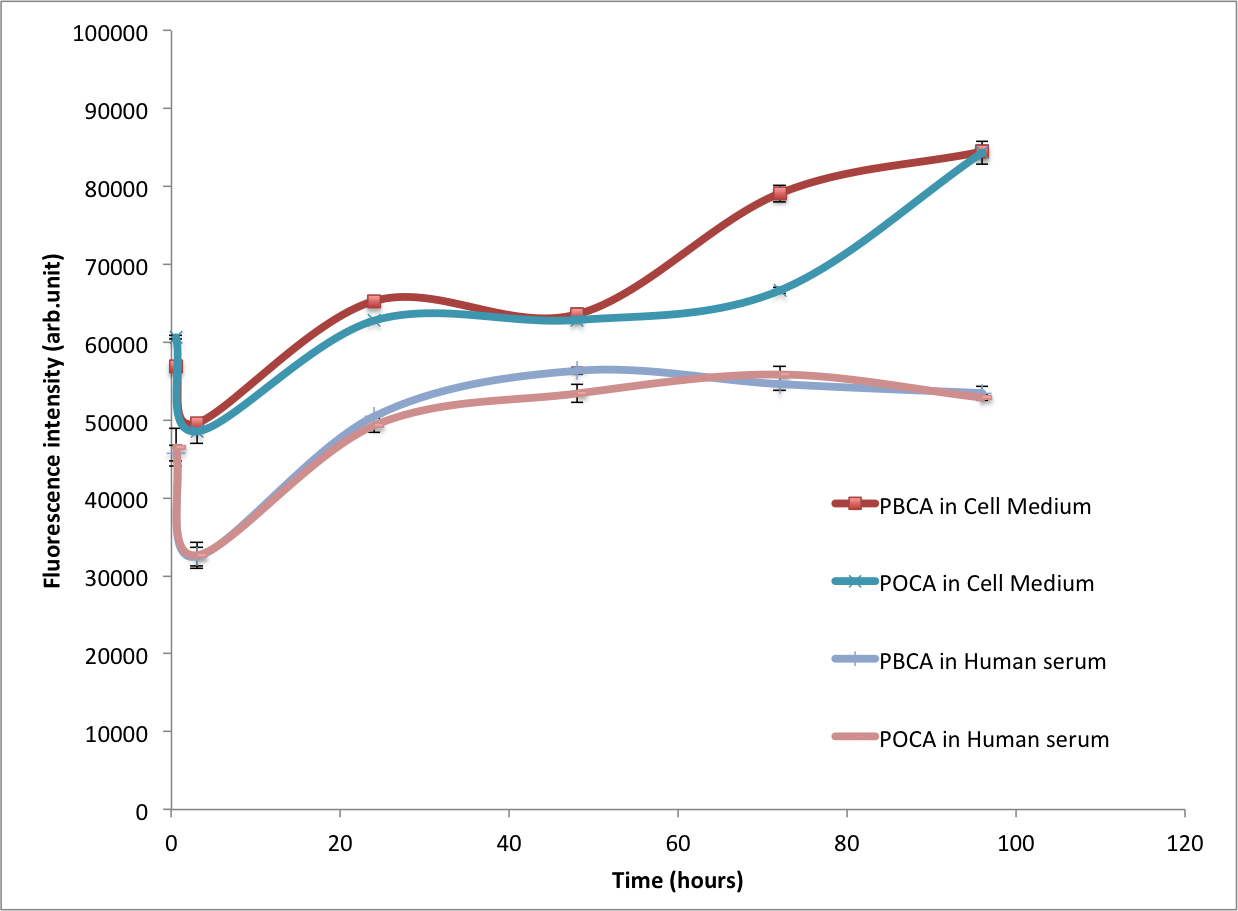


S3: The esterase activity in cell medium and human serum with PBCA and POCA NPs. Each data point are the mean of 3 measurements, bar indicate standard deviation SD. Complete hydrolysis gave fluorescence values of 95000.
